# Effect of Transcranial Direct Current Stimulation (tDCS) on Depression in Parkinson’s Disease—A Narrative Review

**DOI:** 10.3390/jcm13030699

**Published:** 2024-01-25

**Authors:** James Chmiel, Filip Rybakowski, Jerzy Leszek

**Affiliations:** 1 Institute of Neurofeedback and tDCS Poland, 70-393 Szczecin, Poland; 2 Department and Clinic of Psychiatry, Poznan University of Medical Sciences, 61-701 Poznań, Poland; 3 Department and Clinic of Psychiatry, Wrocław Medical University, 54-235 Wrocław, Poland

**Keywords:** tDCS, transcranial direct current stimulation, Parkinson’s disease, non-invasive brain stimulation, neurostimulation, neuromodulation

## Abstract

Introduction: Depression is the most prevalent comorbid neuropsychiatric condition in individuals with Parkinson’s disease (PD), and its underlying mechanisms are not yet fully understood. Current treatment methods are characterised by moderate effectiveness and possible side effects, prompting the search for new non-invasive and safe treatment methods. Methods: This narrative review explores the use of transcranial direct current stimulation (tDCS) in the treatment of depression in PD, based on neuropsychological measures. Searches were conducted in the PubMed/Medline, Research Gate, and Cochrane databases. Results: Nine relevant studies were identified, where depression scores served as either primary or secondary outcomes. Stimulation protocols displayed heterogeneity, especially concerning choice of stimulation site. Patient samples were also heterogeneous. The majority of the studies incorporated anodal stimulation targeting the left dorsolateral prefrontal cortex (DLPFC). The results revealed a reduction in depression scores among PD patients following tDCS. Potential mechanisms through which tDCS may alleviate depression in PD were discussed and recommendations for future research were made. Conclusions: Preliminary evidence suggests that tDCS applied anodally to the left DLPFC reduces depression scores in people with PD; however, due to the heterogeneity of the studies analysed, the use of tDCS in this field should be approached with caution and warrants further validation and confirmation.

## 1. Introduction

Parkinson’s disease (PD) is a common neurodegenerative disorder with an incidence that increases with age, affecting approximately 1% of the population [1]. The disease is characterised by the progressive loss of dopaminergic neurons and the accumulation of α-synuclein aggregates, leading to various motor symptoms such as muscle stiffness, slowness of movement, tremors, and postural instability [2,3]. Although PD is primarily recognised as a movement disorder, it involves several non-motor symptoms that extend beyond motor skills. These include sleep disorders, sensory disturbances, behavioural changes, autonomic dysfunctions, and depression [4]. Depression, in particular, is a significant neuropsychiatric symptom that occurs throughout all stages of PD [5]. It is considered the most common symptom according to the Non-Motor Symptoms Scale (NMSS) and significantly impacts the quality of life of individuals with PD [6,7,8,9]. Depression in PD is characterised by deep feelings of sadness, a sense of hopelessness, reduced energy levels, diminished motivation, and a loss of interest in life [10]. Notably, depression symptoms often precede the onset of motor symptoms and may even be the first manifestation of the disease, occurring up to five years before motor symptoms emerge [11,12,13].

As previously mentioned, depression is one of the most common non-motor symptoms in in PD, but determining the exact number of affected patients can be challenging due to variations in validation, definitions, and sample diversity. According to Jellinger [14], the estimated prevalence of depression in PD ranges widely, ranging from 2.7% to 90% with a mean of 38%. Surprisingly, only about 26% of PD patients receive treatment for depression [5]. Unfortunately, clinical depression in PD is often underdiagnosed and inadequately treated, partly due to concerns about the side effects of medications used for motor symptoms [15,16]. This underscores the importance of early diagnosis, as initiating treatment promptly can positively impact the effectiveness of PD management, disease burden, disability, and cognitive decline [14,17].

The underlying mechanisms of depression in PD are not fully understood, but several factors have been identified. One key factor is the dysfunction in the production of neurotransmitters in the basal ganglia, specifically dopamine, serotonin, and norepinephrine [18]. PD patients experience degeneration of the nigrostriatal dopaminergic pathway due to a pronounced loss of neurons in the substantia nigra pars compacta (SNc), locus coeruleus, and dorsal raphe nuclei [14]. Structural changes in the brain are also observed in PD-related depression, including limbic and striatal atrophy, lower grey and white matter density in various regions, and disruption of mood-related network connections. Other potential factors contributing to depression in PD include metabolic disorders, neuroinflammation, immunological reactions, dysregulation of the hypothalamic–pituitary–adrenal (HPA) axis, abnormal hippocampal neurogenesis, dysregulation of trophic support, and genetic and vascular factors [14,19]. For a more comprehensive understanding of the precise mechanisms involved in depression in PD, please refer to recent review articles [7,14,19,20].

Various approaches exist for treating depression in PD. Pharmacotherapy is commonly employed as a first-line treatment. Several classes of antidepressant drugs have demonstrated efficacy and safety, including tricyclic antidepressants (TCAs), selective serotonin reuptake inhibitors (SSRIs), and non-selective monoamine oxidase inhibitors (MAOIs) [21,22,23,24]. Specific drugs such as venlafaxine, citalopram, and paroxetine have been frequently prescribed [20]. Another therapeutic avenue involves drugs that target dopamine receptors. For instance, Bupropion is commonly administered because it can modulate both the dopaminergic and noradrenergic systems. By inhibiting the reuptake of these neurotransmitters and preserving their innervation in the limbic system, bupropion helps alleviate depressive symptoms [19,25]. Behavioural interventions, including cognitive-behavioural therapy, may also be beneficial as adjunctive treatments for depression in PD, although research in this area is relatively limited [20,26]. Additionally, engaging in physical activity has shown positive effects on the well-being of individuals with PD. It can increase dopaminergic release in the caudate nucleus and improve mesolimbic functioning [7,27].

In conclusion, depression in PD is a multifaceted issue that significantly impacts the well-being of patients. Researchers are actively exploring additional non-invasive and safe treatment approaches, and one promising avenue is transcranial direct current stimulation (tDCS), which falls within the category of non-invasive brain stimulation (NIBS) techniques.

The tDCS technique involves the application of a low voltage and current (typically 1–2 mA) through two electrodes, an anode and a cathode, positioned on the head [28]. A battery-powered stimulator delivers the electrical current, with a portion reaching the brain. Stimulation induces a general pattern of increased cortical excitability beneath the anode and decreased excitability beneath the cathode (the reference/return electrode), which can persist for up to 90 min after stimulation [29]. Specifically, tDCS works by modulating the firing rate of neurons through changes in the neuronal membrane potential within the targeted brain region [30]. Anodal tDCS depolarises neurons, facilitating their firing, while cathodal tDCS hyperpolarises neurons, inhibiting their firing [31]. The neuroplastic effects of tDCS are influenced by various stimulation parameters and involve calcium-dependent modulation of synaptic plasticity in *n*-methyl-D-aspartate (NMDA) glutamatergic neurons, similar to mechanisms observed in long-term potentiation (LTP) and long-term depression (LTD) [32]. Overall, tDCS is considered a safe technique with minimal side effects [33].

As a technique, tDCS has been extensively studied in the context of PD and numerous studies have demonstrated its effectiveness in improving various aspects of the disease, such as gait [34], cognitive deficits [35], fatigue [36], and balance and functional mobility [37]. However, it is important to note that the effectiveness of tDCS specifically for treating depression in PD has not been thoroughly investigated.

This narrative review aims to explore the potential applications of tDCS in mitigating the symptoms of depression in PD. By modulating cortical excitability, tDCS has the potential to induce behavioural changes and promote neurofunctional reorganisation. Given that depression in PD is associated with reduced activity in certain brain regions, tDCS treatment may hold promise in addressing this aspect of the disease.

## 2. Methods

### 2.1. Data Sources and Search Strategy

For this narrative review, J. Ch., F. R., and J. L. performed an independent online search using predefined criteria. The following combined keywords were used: “transcranial direct current stimulation” OR “tDCS” AND “Parkinson” OR “Parkinson’s disease”. We considered publications in the PubMed/Medline, Research Gate, and Cochrane databases, with an access date of May 2023 and publication dates ranging from January 2008 to May 2023.

### 2.2. Study Selection Criteria

Eligibility criteria included clinical trials conducted in English, published from 2008 to 2023. Considered studies investigated the effects of tDCS on depression in Parkinson’s disease as a primary or secondary outcome. The exclusion criteria encompassed articles that were not published in English and reviews.

### 2.3. Screening Process

The screening process was conducted in multiple stages to ensure the inclusion of relevant studies and the exclusion of those that did not meet the predefined criteria. The initial screening involved a thorough examination of titles and abstracts by the reviewers (J. Ch., F. R., and J. L.), independently.

#### 2.3.1. Title and Abstract Screening

Each reviewer independently assessed the titles and abstracts of retrieved records to identify studies that potentially met the inclusion criteria. During this stage, the screening criteria focused on relevance to transcranial direct current stimulation and its effects on depression in Parkinson’s disease.

#### 2.3.2. Full-Text Assessment

Following the title and abstract screening, the selected articles underwent a comprehensive full-text assessment. Reviewers examined the complete manuscripts to determine if they met the detailed eligibility criteria, emphasizing the inclusion of clinical trials conducted in English and published between January 2008 and May 2023.

## 3. Results

**Figure 1:**
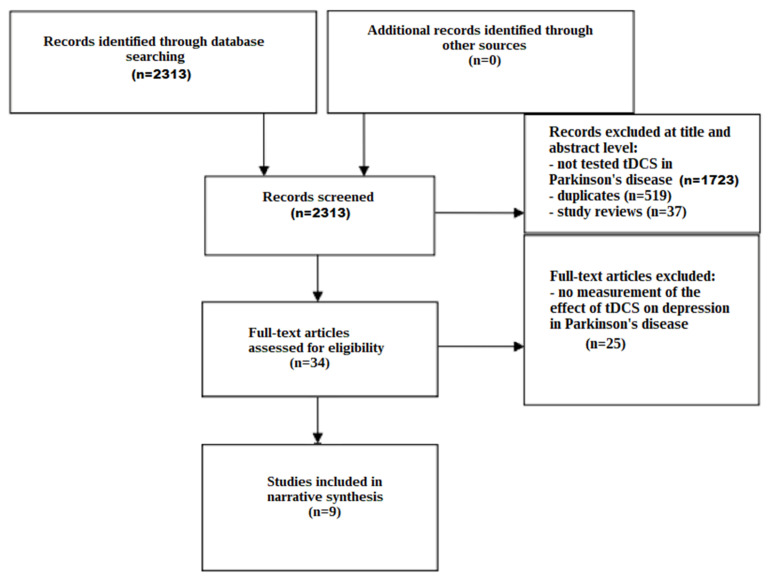
Flow chart depicting the different phases of the systematic review.

The screening process is illustrated in a flow chart (Figure 1). Through the search strategies carried out in the databases, 2313 studies were identified. A total of 2279 studies were excluded based on the evaluation of their titles and abstracts, due to not testing tDCS in Parkinson’s disease (*n* = 1723), due to removal of duplicates (*n* = 519), and due to removal of study reviews (*n* = 37). Afterwards, 34 studies were identified and underwent a comprehensive full-text assessment. Of these, 25 studies were excluded, because they did not measure the effect of tDCS on depression in Parkinson’s disease. After a full reading of the texts, nine articles were deemed eligible for inclusion.

The included studies were published between 2014 and 2022. A total of 200 patients were enrolled (active tDCS = 139, sham tDCS = 61). Among them, five studies were RCTs, two were feasibility studies, one was an open label pilot study, and one was a cross-over study. Random assignment occurred in five of the studies. In six of the studies, sham stimulation was used for the control groups, with patients and researchers being blinded. Typically, the studies employed a current ranging from 1 to 2 mA for tDCS. Regarding the electrode montage, the most commonly utilised approach was the bipolar montage. In this configuration, electrodes were positioned directly on the scalp, and the same current intensity was delivered through both the anode and cathode. In other studies, a monopolar montage was employed, with one electrode on the scalp while the other was positioned extracranially, on areas such as on the deltoid muscle, buccinator muscle, or mastoid.

### 3.1. Summary of Included Studies

The included studies are summarised in Table 1. Hadoush et al. [38] conducted a first study to investigate the effects of bilateral anodal tDCS on melatonin serum levels, sleep functions, and depression in patients with PD. The study included 25 participants diagnosed with idiopathic PD. The level of depression perception was assessed using the Geriatric Depression Scale (GDS). During the tDCS sessions, two anodal electrodes were placed over the left FC1 and right FC2 regions, while two cathodal electrodes were positioned over the left and right Fp1 and Fp2 supraorbital areas. The applied current intensity was 1 mA, and each session lasted for 20 min. Each patient received a total of 10 tDCS sessions.

A second study conducted by Hadoush et al. [39] explored the impact of tDCS on sleep quality, depression, and quality of life in patients with PD. The study included 21 participants with PD who underwent a total of 10 tDCS sessions, each lasting 20 min. During the tDCS sessions, bilateral anodal stimulation was applied simultaneously over the left and right prefrontal (F3) and motor (C3) areas. Additionally, two cathode electrodes were placed over the left and right supraorbital areas. The intensity of the applied current was 1 mA. The level of depression was assessed using the GDS.

In the Benninger et al. [40] study, the effects of tDCS on movement, cognition, and depression in PD were examined. The study involved 25 patients who received either real or sham stimulation. During the tDCS sessions, anodal stimulation was administered at a current intensity of 2 mA for a duration of 20 min. The anodal electrode was placed symmetrically either over the pre-motor or motor areas, with the centre of the electrode positioned 8 mm anterior to Cz, or over the prefrontal cortices (forehead above eyebrows). In each session, a single target area was stimulated, and the position of the anode was alternated between sessions, starting with the motor area, ensuring that each target area was stimulated four times. Cathodal electrodes were positioned over the mastoids. The Beck Depression Inventory (BDI) was utilised to evaluate the level of depression.

Oh et al. [41] conducted a study with the objective of investigating how tDCS affects the central cholinergic system and cortical excitability in individuals primarily diagnosed with the akinetic-rigid type of PD. A total of 18 patients with PD participated in the study. During the experimental sessions, the patients underwent five sessions of anodal tDCS targeting the primary motor cortex (M1) area, specifically on the contralateral side of their dominant hand. The current intensity applied during the tDCS sessions was set at 2 mA, and each session lasted for 20 min. The level of depression was assessed using the BDI.

In the study conducted by Ferrucci et al. [42], the objective was to evaluate the effects of tDCS applied over the cerebellum and motor cortex on motor and cognitive symptoms, as well as levodopa-induced dyskinesias, in patients diagnosed with PD. The study included nine individuals with idiopathic PD. During the five tDCS sessions, bilateral anodal stimulation was administered at a current intensity of 2 mA for a duration of 20 min. For the stimulation of the primary motor cortex (M1), three sponge electrodes were utilized. Two electrodes were placed on the scalp over the motor cortex bilaterally (C3 and C4), while the third electrode was positioned over the right deltoid muscle. The level of depression was measured using the BDI.

The study conducted by Manenti et al. [43] aimed to examine the effects of anodal tDCS combined with physical therapy on motor and cognitive performance in patients with PD. A total of 20 PD patients participated in the study and were assigned to one of two groups: group 1 received anodal tDCS in addition to physical therapy, while group 2 received sham tDCS alongside physical therapy. During the ten sessions, tDCS was administered for a duration of 25 min concurrently with the physical therapy sessions. The anodal electrode was positioned over either the left or right DLPFC, approximately 8 cm frontally and 6 cm laterally from the scalp vertex, using the F3/4 or F7/8 international 10–20 EEG system. The reference electrode was placed on the contralateral supraorbital area. The current intensity used for tDCS was set at 2 mA. The long-term effects of the treatment were evaluated at a 3-month follow-up, assessing clinical, neuropsychological, and motor task performance. The severity of depression was measured using the Beck Depression Inventory-II (BDI-II).

The study conducted by Manenti et al. [44] aimed to examine the effects of tDCS combined with computerized cognitive training (CCT) on cognition and mood disturbances in patients with PD. A total of 22 PD patients participated in the study and were assigned to either the active tDCS plus CCT group or the sham tDCS plus CCT group. The treatment duration was two weeks, with daily sessions of tDCS applied for 25 min during the CCT tasks targeting functions related to the prefrontal cortex. For the active tDCS group, the anodal electrode was positioned over the left DLPFC, approximately 8 cm frontally and 6 cm laterally from the scalp vertex (over F3). The reference electrode was placed on the right supraorbital area. The current intensity used for tDCS was set at 2 mA. Each patient underwent evaluations at a baseline, immediately after the treatment period, and at a 3-month follow-up. Depressive symptoms were assessed using the BDI-II questionnaire.

The study conducted by Doruk et al. [45] aimed to evaluate both the immediate and long-term effects of ten sessions of anodal tDCS targeting the right DLPFC or left DLPFC. The assessment was carried out at the end of the two-week stimulation period and followed up at one month. The participants were divided into two groups, with five individuals receiving anodal tDCS over the right DLPFC (F4) and six individuals receiving anodal tDCS over the left DLPFC (F3). The stimulation sessions lasted for 20 min, and a current intensity of 2 mA was administered. To assess depression, the researchers utilized two measures: the BDI and the Hamilton Rating Scale for Depression (HRSD). The evaluations were conducted immediately after the two-week stimulation period to assess the immediate effects of tDCS. Additionally, a follow-up evaluation was conducted at one month to investigate the sustained effects of the intervention on depressive symptoms.

The objective of the study conducted by Biundo et al. [46] was to evaluate the combined effects of tDCS and cognitive training (CT) on cognition and depression in patients with PD. A total of 24 PD patients were randomly assigned to two groups: one group received a 4-week course of CT along with real tDCS (12 participants), while the other group received CT along with sham tDCS (12 participants). During the tDCS sessions, the stimulation lasted for 20 min, and an intensity of 2 mA was used. The anodal electrode was positioned over the left DLPFC, while the cathodal electrode was placed over the contralateral supraorbital region. To assess depressive symptoms, the BDI-II was employed. Sixteen patients completed the 16-week follow-up session, allowing for the evaluation of long-term effects.

### 3.2. Effects of tDCS on Depression in PD

In the study conducted by Hadoush et al. [38], the findings indicated that bilateral anodal tDCS resulted in a notable reduction in the average depression levels, as measured with the GDS. This decrease in depression levels was found to be statistically significant (*p* = 0.027) and demonstrated a small to moderate effect size (Cohen’s d = 0.42).

In the study conducted by Hadoush et al. [39], a paired *t*-test analysis revealed a statistically significant decrease in the average total score of the GDS after bilateral anodal tDCS, compared to before the tDCS stimulation. The decrease in the GDS score was significant with a *p*-value of 0.016.

In the study by Benninger et al. [40], the results indicated that there were changes observed in the BDI scores from baseline to post-intervention measurements. However, these changes did not show significant differences between the treatment groups.

The study conducted by Oh et al. [41] found a significant improvement in the BDI scores after anodal stimulation compared to the pre-tDCS measurements. The mean change in BDI scores was 2.67 ± 3.48, and this improvement was statistically significant with a *p*-value of less than 0.01.

The study conducted by Ferrucci et al. [42] showed no significant changes in the BDI scores. The analysis indicated that the BDI scores remained unchanged, with a *p*-value of 0.36, suggesting no statistically significant difference in depression levels following the intervention.

The study conducted by Manenti et al. [43] revealed a significant main effect of time on the BDI-II scores, indicating a reduction in depressive symptoms among the participants. Post hoc analysis further demonstrated a statistically significant decrease in BDI-II scores, reflecting a reduction in depressive symptoms immediately after the treatment. Specifically, the baseline BDI-II score was 11.65 (standard deviation [SD] 6.5), which decreased to 8.95 (SD 6.3) post-treatment. This reduction in depressive symptoms was sustained at the 3-month follow-up, with a BDI-II score of 8.40 (SD 7.5).

In the study conducted by Manenti et al. [44], post hoc analysis revealed a significant decrease in BDI-II scores, specifically in the group receiving anodal tDCS combined with CCT. The analysis showed a significant reduction in BDI-II scores from the baseline to the post-treatment assessment (*p* = 0.048) and from the baseline to the 3-month follow-up (*p* = 0.015).

In the study of Doruk et al. [45], the results indicated that the group receiving anodal tDCS over the left DLPFC showed a greater reduction in BDI scores compared to the sham stimulation group and the group receiving tDCS over the right DLPFC. The mean percentage reduction in BDI scores was −49.8% ± 13.82 in the left DLPFC group, −1.28% ± 11.34 in the sham group, and −22.1% ± 29.82 in the right DLPFC group at the end of the stimulation period.

In the study conducted by Biundo et al. [46], the results indicated that the participants experienced a reduction in BDI-II scores. Specifically, the BDI-II scores dropped by an average of 7 (SD = 8.44) after the intervention period. During the 16-week follow-up, the BDI-II scores further decreased by an average of 4.22 (SD = 13.13).

### 3.3. Other Interventions and Medications Used during tDCS

All PD patients included in the analysed studies were treated with levodopa and/or dopamine agonists. In the study by Ferrucci et al. [42], the patients additionally took various psychotropic drugs, including antidepressants. In the study by Biundo et al. [46], there is no information about medications taken. In the study by Manenti et al. [43], patients participated in physical therapy, and in the studies by Manenti et al. [44] and Biundo et al. [46], patients participated in cognitive training.

### 3.4. Durability of tDCS Effects

Four studies measured the effect of tDCS on depression outcomes over follow-up periods. In the study by Benninger et al. [40], there was a further decrease in depression scores 1 month after the intervention, but there was a slight increase after 3 months. In the study by Manenti et al. [43], improvement in depression was evident in the 3-month follow-up period. In the study by Manenti et al. [44], also, the improvement in depression was maintained after 3 months of observation. In the study by Biundo et al. [46], the improvement in depression was maintained after 12 weeks of follow-up.

## 4. Results

### 4.1. General Findings

Depression is the most prevalent psychiatric disorder associated with Parkinson’s disease. Its severity has a significant impact on prognosis and the effectiveness of treatment. Ongoing efforts are dedicated to finding new therapeutic methods to help patients. This narrative review explored the potential of tDCS to improve depression scores on various neuropsychological scales. Nine studies were included in the analysis and eight of them reported an improvement in depression scores. However, recommending the use of tDCS for depression comorbid with PD is still premature for several reasons.

First, the protocols used were heterogeneous. Various areas of the brain were stimulated, including the left and right DLPFC, M1, FC1, and FC2. Stimulation of the motor cortex has been applied to influence motor parameters in PD, which has been shown to be effective in previous studies. Stimulation of the left DLPFC is understandable because of this area’s involvement in depression. Moreover, stimulation of the left DLPFC is a well-established protocol in the treatment of depression in many studies [47]. The effect of M1 stimulation on depression is unknown. It is possible that there is a network effect of tDCS in this case, as this stimulation is not focused, and placing the electrode contralaterally on the forehead directs some of the current to the prefrontal cortex.

Second, the sample of PD patients was heterogeneous in terms of depression severity and reported improvement in outcomes. In some studies, depression was diagnosed based on measurements, and while tDCS reduced their scores, some patients remained depressed after treatment. In other studies, some patients had higher scores, but those scores did not necessarily indicate depression. Similarly, in this case, tDCS lowered their scores.

Third, all patients were concurrently taking medications that may affect depression, making it not possible to accurately assess the isolated effects of tDCS. However, discontinuing medications in studies involving PD patients is neither safe nor ethical. On the other hand, it was confirmed that tDCS in combination with antidepressants is more effective than when used alone [48].

Despite these limitations, tDCS, especially when targeted at the left DLPFC, appears to be a promising treatment for depression in PD. Nonetheless, it necessitates further validation and confirmation. Potential mechanisms of action are detailed below.

### 4.2. Mechanisms of Action of tDCS in Depression in PD

#### 4.2.1. Impact on Cortical Regions

The underlying hypothesis regarding the use of tDCS in the treatment of depression is that it is linked to dysfunction in various cortical and subcortical regions. Specifically, these regions include the prefrontal cortex [49], the amygdala [50], and the hippocampus [51]. In PD patients with depression, cortical thinning in the prefrontal cortex has been observed [52]. Moreover, depressed individuals often exhibit hemispheric imbalances characterised by increased activity in the right cortex and decreased activity in the left cortex. The fundamental effect of tDCS is to enhance activity in the left dorsolateral prefrontal cortex while reducing activity in the right cortex [53]. By modulating cortical activity in this manner, tDCS aims to address the underlying neurophysiological imbalances associated with depression.

#### 4.2.2. Neuroplasticity Enhancement

Individuals with depression often show decreased neuroplasticity in the motor cortex when subjected to paired-associative stimulation (PAS) [54]. This implies that neuroplasticity is impaired in depression [55]. A study conducted by Player et al. [56] demonstrated that anodal stimulation of the left DLPFC using tDCS increased PAS-induced neuroplasticity in the motor cortex. This finding suggests that tDCS has the potential to enhance neuroplasticity in individuals with depression. By facilitating neuroplastic changes, tDCS may contribute to the amelioration of depressive symptoms and the restoration of neural functioning in affected individuals.

#### 4.2.3. Brain-Derived Neurotrophic Factor (BDNF) Modulation

One hypothesis suggests that depression arises when neuroplasticity decreases as a result of deficits in the neurotrophic support provided by brain-derived neurotrophic factor (BDNF) [57]. BDNF plays a crucial role in neuronal survival and synaptic potentiation [58], and its involvement in the development of depressive disorders and response to antidepressant treatments has been well-recognised [59]. In the context of PD, low levels of BDNF have been observed, irrespective of the presence of depression [60]. Emerging evidence suggests that reduced BDNF levels may specifically contribute to the development of depression in PD [61], and they potentially serve as a discriminative marker between depressed and non-depressed PD patients [62]. A meta-analysis by Irwin et al. [63] demonstrated that non-invasive brain stimulation techniques, including tDCS, significantly increase the expression of BDNF compared to sham stimulation. This upregulation of BDNF has been associated with improved depression scores, highlighting the potential of tDCS and other NIBS methods in the treatment of depression.

#### 4.2.4. Top-Down Network Modulation

Depression is characterised by dysfunction in the top–down network responsible for effortful emotion regulation (ER), involving the dorsal and ventromedial prefrontal cortex (vmPFC) and the amygdala. In a study utilizing fMRI neuroimaging, Chrysikou et al. [64] demonstrated that stimulation of the left DLPFC leads to increased activity in the medial prefrontal cortex (MPFC) and can impact reappraisal performance by modulating the functional connectivity between the MPFC and the bilateral amygdala. Specifically, left DLPFC stimulation was found to weaken the functional connectivity between the MPFC and the amygdala, potentially influencing emotional regulation processes.

#### 4.2.5. Dopaminergic System Modulation

The development of depression in PD is associated with the progressive loss of dopaminergic neurons in the nigrostriatal pathway [19]. PD leads to a depletion of dopaminergic neurons in the substantia nigra (SN), which is believed to contribute to the pathophysiology of depression in PD. Furthermore, there is evidence of reduced availability of the dopamine transporter (DAT), indicating presynaptic dopaminergic dysfunction. The deficiency of dopamine can disrupt the reward system and result in anhedonia, a key symptom of depression [14]. Research suggests that tDCS may have the ability to modulate the release of monoamine transmitters, including dopamine, in brain circuits that are distal to the stimulation sites [65,66]. Stimulation of the left DLPFC has been shown to increase dopamine release in the right ventral striatum, which plays a role in reward processing [67]. By influencing the dopaminergic system, tDCS may have a potential role in ameliorating depressive symptoms in PD.

#### 4.2.6. Glutamatergic Neurotransmission Modulation

Lower levels of glutamate, the primary excitatory neurotransmitter in the brain, have been observed in the cerebral cortex of individuals with depression [68]. Both ionotropic receptors (NMDA, AMPA) and metabotropic receptors (mGluR) for glutamate play a role in mood modulation and have been found to be dysfunctional in depression [69]. This has prompted the development of treatments targeting AMPA and NMDA receptors to address depressive symptoms [70]. A study by Alvarez-Alvarado et al. [71] investigated the effects of tDCS on the stimulation on glutamine/glutamate (Glx) and gamma-aminobutyric acid (GABA) levels. Specifically, tDCS stimulation of the left DLPFC revealed an increase in Glx concentrations from pre- to post-intervention, suggesting that tDCS may have an impact on glutamatergic neurotransmission. This modulation of Glx levels may have implications for the treatment of depression.

#### 4.2.7. Anti-Inflammatory Effects

There is evidence suggesting that neuroinflammation plays a role in depression in PD [19,72], leading to neuronal loss [73]. Neuroinflammation, a characteristic pathological feature of PD [74], is associated with increased pro-inflammatory factors [14]. Studies consistently demonstrate elevated levels of pro-inflammatory cytokines, such as interleukin-6 (IL-6) and tumour necrosis factor alpha (TNF-α) [14], in the brains and blood of individuals with PD and depression [14,75,76]. In the context of tDCS, several studies have explored its potential to modulate neuroinflammation. Guo et al. [77] conducted an animal study using anodal tDCS and observed a reduction in the expression of pro-inflammatory cytokines, including IL-1β, IL-6, and TNF-α, leading to a decrease in the inflammatory response in the hippocampus. In two subsequent studies by Brunoni et al. [78,79], tDCS was applied to depressed patients with anodal stimulation of the left DLPFC and cathodal stimulation of the right DLPFC. Plasma levels of various cytokines, including IL-6, IL-10, TNF-α, and IL-1β, were measured before and after tDCS, and all cytokine levels were found to decrease following tDCS. Another study by Goerigk et al. [80] focused on patients with bipolar depression and measured the levels of interleukins (IL-2, 4, 6, 8, 10, 18, 33, 1β, 12p70, 17a), interferon gamma (IFN), and TNF-α. They reported a decrease in IL-8 levels after tDCS. These findings suggest that tDCS may have anti-inflammatory effects by modulating the production of pro-inflammatory cytokines.

#### 4.2.8. Lowering α-Synuclein Levels

The role of alpha-synuclein (α-Syn) in depression in PD is connected to its impact on the serotonin (5-HT) system [81]. α-Syn aggregates, a major component of Lewy pathology [82], are found in neuronal populations, including cholinergic, norepinephrine, and serotonin neurons [83], contributing to non-motor symptoms. Dysfunction of the 5-HT system has been implicated in anxiety and depressive disorders [81]. Recent research showed the impaired integrity of the 5-HT system in PD, suggesting a specific causal role in the progression of various PD symptoms like depression [81]. Neuropathological studies have shown the presence of Lewy bodies in raphe 5-HT neurons [84,85], indicating α-Syn-related pathology in the early stages of PD. Other works report neuronal loss in the raphe nuclei, while others emphasise morphological changes in 5-HT fibres [81]. These alterations contribute to modified 5-HT neurotransmission, potentially leading to depressive symptoms [81]. In [86], research conducted in a human neuroblastoma cell line under basal conditions showed that tDCS effectively reversed the accumulation of α-Syn, not by influencing the gene transcription of α-Syn, but by enhancing its degradation. In a different experiment using a mouse model induced with 1-methyl-4-phenyl-1,2,3,6-tetrahydropyridine (MPTP) to simulate PD, tDCS was found to decrease the levels of α-synuclein protein [87]. In summary, both studies suggest that tDCS can influence the serotonin system through its inhibitory effect on α-synuclein, directly or indirectly influencing the symptoms of depression in PD.

### 4.3. Future Perspectives

There is an urgent need to conduct new, high-quality RCTs with as large sample sizes as possible to confidently confirm the antidepressant effect of tDCS in PD (for review see [88,89]). It is crucial that the reduction of depression is a primary outcome, and that depression should be validated by a psychiatrist using widely accepted and validated neuropsychological measures to avoid misinterpretation of the results. To ensure comparability between new and existing studies, we suggest using the BDI-II and HRSD to measure depression in people with PD. Additionally, the assessment can be supplemented with Non-Motor Symptoms Scale (NMSS) measurements. Future studies should create control groups that use medications, psychotherapy, or other interventions alone or in combination with tDCS. Evidence shows that tDCS, when combined with other treatment options, is more effective in treating depression than individual interventions used alone [48]. It is also important to assess the progression of PD, estimate the time since diagnosis of PD and depression, and select patients into groups based on similar disease outcomes and demographics (similar age). The left DLPFC seems to be the appropriate site of stimulation for further research. Other measurements, such as fMRI and EEG, should be incorporated to capture the mechanisms of action of the stimulation. The effect of tDCS on α-synuclein levels can be examined, but it is also worth testing other biomarkers such as TNF-α [90] and IL-17A [91].

## 5. Conclusions

In conclusion, the current evidence on the effectiveness of tDCS in the treatment of depression in PD remains uncertain, and stimulation cannot be recommended as a therapeutic method. However, the results from existing studies are encouraging and suggest the potential for tDCS as a treatment option. To definitively confirm or disprove the efficacy of neuromodulation tDCS in the treatment of depression in PD, future clinical trials are required.

## Figures and Tables

**Table 1:** Summary of main findings from articles included in the review.

Author, Citation	Population	Used Test	Interventions	Stimulation Site	Current Intensity	Duration (min)	Main Findings in Treatment Group	Follow-Up
Hadoush et al. [38]	25 patients	GDS	10 sessions	Two anodal electrodes over the left FC1 and right FC2, two cathodal electrodes over the left and right Fp1 and Fp2 supraorbital areas.	1 mA	20	Reduction in the average depression levels, as measured using the GDS. The decrease in depression levels was found to be statistically significant (*p* = 0.027), with a small to moderate effect size (Cohen’s d = 0.42).	
Hadoush et al. [39]	21 patients	GDS	10 sessions	Bilateral anodal stimulation was applied simultaneously over the left and right prefrontal (F3) and motor (C3) areas. Two cathode electrodes were placed over the left and right supraorbital areas.	1 mA	20	Statistically significant decrease in the average total score of the GDS.	
Benninger et al. [40]	25 patients 13 real tDCS 12 sham tDCS	BDI	Each target area was stimulated four times (eight sessions)	The anodal electrode was placed symmetrically either over the pre-motor or motor areas, with the center of the electrode positioned 8 mm anterior to Cz, or over the prefrontal cortices (forehead above eyebrows). Cathodal electrodes were positioned over the mastoids.	2 mA	20	The results indicated that there were changes observed in the BDI scores from baseline to post-intervention measurements. However, these changes did not show significant differences between the treatment groups.	There was a further decrease in depression scores 1 month after the intervention, but there was a slight increase after 3 months.
Oh et al. [41]	18 patients	BDI	Five sessions	Anodal tDCS targeting the primary motor cortex (M1) area, specifically on the contralateral side of their dominant hand.	2 mA	20	Significant improvement in the BDI scores after anodal stimulation compared to before. The mean change in BDI scores was 2.67 ± 3.48, and this improvement was statistically significant with a *p*-value of less than 0.01.	
Ferrucci et al. [42]	Nine patients	BDI	Five sessions	For the anodal stimulation of the primary motor cortex (M1), three sponge electrodes were utilized. Two electrodes were placed on the scalp over the motor cortex bilaterally (C3 and C4), while the third electrode was positioned over the right deltoid muscle.	2 mA	20	No significant changes in the BDI scores.	
Manenti et al. [43]	20 patients 10 real tDCS 10 sham tDCS	BDI-II	10 sessions	The anodal electrode was positioned over either the left or right DLPFC, approximately 8 cm frontally and 6 cm laterally from the scalp vertex, using the F3/4 or F7/8 international 10–20 EEG system. The reference electrode was placed on the contralateral supraorbital area.	2 mA	25	Statistically significant decrease in BDI-II scores.	Improvement in depression was evident in the 3-month follow-up period.
Manenti et al. [44]	22 patients	BDI-II	10 sessions	Anodal electrode was positioned over the left DLPFC, approximately 8 cm frontally and 6 cm laterally from the scalp vertex (over F3). The reference electrode was placed on the right supraorbital area.	2 mA	25	The analysis showed a significant reduction in BDI-II scores from baseline to the post-treatment assessment.	The improvement in depression was maintained after 3 months of observation.
Doruk et al. [45]	11 patients	BDI, HRSD	10 sessions	Anodal tDCS over the right DLPFC (F4) or anodal tDCS over the left DLPFC (F3).	2 mA	20	A greater reduction in BDI scores compared to the sham stimulation group and the group receiving tDCS over the right DLPFC. The mean percentage reduction in BDI scores was −49.8% ± 13.82 in the left DLPFC group.	
Biundo et al. [46]	24 patients 12 real tDCS 12 sham tDCS	BDI-II	16 sessions	The anodal electrode was positioned over the left DLPFC, while the cathodal electrode was placed over the contralateral supraorbital region.	2 mA	20	The results indicated that the participants experienced a reduction in BDI-II scores. Specifically, the BDI-II scores dropped by an average of 7 (SD = 8.44) after the intervention period.	During the 16-week follow-up, the BDI-II scores further decreased by an average of 4.22 (SD = 13.13).

## Data Availability

No new data were created or analysed in this study. Data sharing is not applicable to this article.
